# Neoadjuvant Chemoradiotherapy and Surgery for Esophageal Squamous Cell Carcinoma Versus Definitive Chemoradiotherapy With Salvage Surgery as Needed: The Study Protocol for the Randomized Controlled NEEDS Trial

**DOI:** 10.3389/fonc.2022.917961

**Published:** 2022-07-13

**Authors:** Magnus Nilsson, Halla Olafsdottir, Gabriella Alexandersson von Döbeln, Fernanda Villegas, Giovanna Gagliardi, Mats Hellström, Qiao-Li Wang, Hemming Johansson, Val Gebski, Jakob Hedberg, Fredrik Klevebro, Sheraz Markar, Elizabeth Smyth, Pernilla Lagergren, Ghazwan Al-Haidari, Lars Cato Rekstad, Eirik Kjus Aahlin, Bengt Wallner, David Edholm, Jan Johansson, Eva Szabo, John V. Reynolds, CS Pramesh, Naveen Mummudi, Amit Joshi, Lorenzo Ferri, Rebecca KS Wong, Chris O’Callaghan, Jelena Lukovic, Kelvin KW Chan, Trevor Leong, Andrew Barbour, Mark Smithers, Yin Li, Xiaozheng Kang, Feng-Ming Kong, Yin-Kai Chao, Tom Crosby, Christiane Bruns, Hanneke van Laarhoven, Mark van Berge Henegouwen, Richard van Hillegersberg, Riccardo Rosati, Guillaume Piessen, Giovanni de Manzoni, Florian Lordick

**Affiliations:** ^1^ Division of Surgery, Department of Clinical Science, Intervention and Technology, Karolinska Institutet, Stockholm, Sweden; ^2^ Department of Upper Abdominal Diseases, Karolinska Comprehensive Cancer Center, Karolinska University Hospital, Stockholm, Sweden; ^3^ Thoracic Oncology Center, Karolinska Comprehensive Cancer Center, Karolinska University Hospital, Stockholm, Sweden; ^4^ Section of Radiotherapy Physics and Engineering, Department of Medical Radiation Physics and Nuclear Medicine, Karolinska Comprehensive Cancer Center, Karolinska University Hospital, Stockholm, Sweden; ^5^ Center for Clinical Cancer Studies, Karolinska Comprehensive Cancer Center, Karolinska University Hospital, Stockholm, Sweden; ^6^ Department of Oncology-Pathology, Karolinska Institutet, Stockholm, Sweden; ^7^ NHMRC Clinical Trials Centre, University of Sydney, Sydney, NSW, Australia; ^8^ Department of Surgical Sciences, Uppsala University Hospital, Uppsala, Sweden; ^9^ Nuffield Department of Surgery, University of Oxford, Oxford, United Kingdom; ^10^ Department of Molecular Medicine and Surgery, Karolinska Institutet, Stockholm, Sweden; ^11^ Department of Oncology, Cambridge University Hospitals National Health Service Foundation Trust, Cambridge, United Kingdom; ^12^ Department of Surgery and Cancer, Imperial College London, London, United Kingdom; ^13^ Department of Oncology, Oslo University Hospital, Oslo, Norway; ^14^ Department of Gastrointestinal Surgery, St. Olav’s Hospital, Trondheim University Hospital, Trondheim, Norway; ^15^ Department of GI and HPB Surgery, University Hospital of Northern Norway, Tromsø, Norway; ^16^ Department of Surgical and Perioperative Sciences, Surgery, Umeå University, Umeå, Sweden; ^17^ Department of Surgery, Linköping University Hospital, Linköping, Sweden; ^18^ Department of Surgery, Skåne University Hospital, Lund, Sweden; ^19^ Department of Surgery, University Hospital of Örebro, Örebro, Sweden; ^20^ Department of Surgery, Trinity St James’s Cancer Institute, Dublin, Ireland; ^21^ Tata Memorial Centre, Homi Bhabha National Institute, Mumbai, India; ^22^ Department of Thoracic Surgery, McGill University Health Centre, Montreal, QC, Canada; ^23^ Radiation Medicine Program, Princess Margaret Cancer Center, University Health Network, Toronto, ON, Canada; ^24^ Department of Radiation Oncology, University of Toronto, Toronto, ON, Canada; ^25^ Canadian Cancer Trials Group, Kingston, ON, Canada; ^26^ Sunnybrook Health Sciences Centre, University of Toronto, Toronto, ON, Canada; ^27^ Sir Peter MacCallum Department of Oncology, Peter MacCallum Cancer Centre, University of Melbourne, Melbourne, VIC, Australia; ^28^ Academy of Surgery, University of Queensland, Princess Alexandra Hospital, Brisbane, QLD, Australia; ^29^ Department of Thoracic Surgery, National Cancer Center/National Clinical Research Center for Cancer/Cancer Hospital, Chinese Academy of Medical Sciences and Peking Union Medical College, Beijing, China; ^30^ Thoracic Oncology Center, HKU Shenzhen Hospital, Hong Kong University Li Ka Shing Medical School, Shenzhen, China; ^31^ Department of thoracic surgery, Chang Gung Memorial Hospital-Linkou, Chang Gung University, Taoyuan, Taiwan; ^32^ Department of Clinical Oncology, Velindre Cancer Centre, Cardiff, United Kingdom; ^33^ Department of General, Visceral and Cancer Surgery, University Hospital of Cologne, Cologne, Germany; ^34^ Department of Medical Oncology, Cancer Center Amsterdam, Amsterdam University Medical Centers, Amsterdam, Netherlands; ^35^ Department of Surgery, Cancer Center Amsterdam, Amsterdam University Medical Centers, University of Amsterdam, Amsterdam, Netherlands; ^36^ Departmentof Surgery, University Medical Center Utrecht, Utrecht, Netherlands; ^37^ Department of Gastrointestinal Surgery, San Rafaele Hospital, Vita Salute University, Milan, Italy; ^38^ Univ. Lille, CNRS, Inserm, CHU Lille, UMR9020-U1277 - CANTHER – Cancer Heterogeneity Plasticity and Resistance to Therapies, F-59000 Lille, France; ^39^ Upper GI Surgery Division, University of Verona, Verona, Italy; ^40^ University Cancer Center Leipzig, Leipzig University Medical Center, Leipzig, Germany

**Keywords:** esophageal squamous cell carcinoma, neoadjuvant chemoradiotherapy, definitive chemoradiotherapy, locoregional surveillance, salvage esophagectomy

## Abstract

**Background:**

The globally dominant treatment with curative intent for locally advanced esophageal squamous cell carcinoma (ESCC) is neoadjuvant chemoradiotherapy (nCRT) with subsequent esophagectomy. This multimodal treatment leads to around 60% overall 5-year survival, yet with impaired post-surgical quality of life. Observational studies indicate that curatively intended chemoradiotherapy, so-called definitive chemoradiotherapy (dCRT) followed by surveillance of the primary tumor site and regional lymph node stations and surgery only when needed to ensure local tumor control, may lead to similar survival as nCRT with surgery, but with considerably less impairment of quality of life. This trial aims to demonstrate that dCRT, with selectively performed salvage esophagectomy only when needed to achieve locoregional tumor control, is non-inferior regarding overall survival, and superior regarding health-related quality of life (HRQOL), compared to nCRT followed by mandatory surgery, in patients with operable, locally advanced ESCC.

**Methods:**

This is a pragmatic open-label, randomized controlled phase III, multicenter trial with non-inferiority design with regard to the primary endpoint overall survival and a superiority hypothesis for the experimental intervention dCRT with regard to the main secondary endpoint global HRQOL one year after randomization. The control intervention is nCRT followed by preplanned surgery and the experimental intervention is dCRT followed by surveillance and salvage esophagectomy only when needed to secure local tumor control. *A target sample size of 1200 randomized patients is planned in order to reach 462 events (deaths) during follow-up.*

**Clinical Trial Registration:**

www.ClinicalTrials.gov, identifier: NCT04460352.

## Introduction

Currently, esophageal cancer is the 7^th^ most common cancer and the 6^th^ most common cause of cancer deaths worldwide (1). Most esophageal cancers are diagnosed in advanced stages. Esophageal squamous cell carcinoma (ESCC) comprises more than 90% of cases of esophageal cancer worldwide, and despite the recent years’ decrease in some Western countries, there are no signs of a global decrease in incidence (1).

Treatment with curative intent for locally advanced ESCC is controversial. Surgical resection of the esophagus was the first established treatment (2). Another alternative is definitive chemoradiotherapy (dCRT), which was first established in the early 1990s (3). Survival after surgical resection alone and after dCRT alone are similar, with five-year overall survival of around 25-30% (4, 5). A third curative treatment approach is esophagectomy preceded by neoadjuvant chemoradiotherapy (nCRT), which was shown to be superior to surgery alone in the CROSS trial, using a novel neoadjuvant chemoradiotherapy regimen consisting of weekly carboplatin and paclitaxel and a total radiation dose of 41.4 Gy, followed by esophagectomy (4). The CROSS trial showed an extraordinary 5-year overall survival after nCRT and surgery of 60% for ESCC, compared to only 33% for surgery alone (6). These outcomes after nCRT followed by planned surgery established the CROSS nCRT regimen with subsequent esophagectomy as a new standard of care for ESCC.

Importantly, observational studies indicate that dCRT, supplemented with salvage esophagectomy in cases of incomplete response or subsequent local recurrence, i.e. when needed for local tumor control, can reach survival levels equivalent to those seen with nCRT with subsequent mandatory esophagectomy (7, 8). The great advantage with this approach is that more than half of the patients will not need to undergo surgery and may consequently be able to avoid the deterioration of health-related quality of life (HRQOL) known to be associated with esophagectomy (9, 10). Based on these studies both nCRT with surgery, as well as dCRT with surveillance and salvage surgery when needed, are recommended treatment options for locally advanced ESCC in the European Society for Medical Oncology (ESMO) guidelines (11), although in many countries a majority of patients with operable ESCC are currently treated with nCRT and surgery, due to the more robust evidence available for this treatment (12–14).

To date, no adequately powered randomized trial comparing these two treatment alternatives has been published, or to our knowledge even been started or planned. The only two previous randomized trials addressing comparable research questions, both with recruitment in the time period around two decades ago, are today obsolete with regard to the compared interventions, both surgery and chemoradiotherapy. Furthermore, these did not include structured locoregional recurrence surveillance after dCRT (15, 16). In addition both these trials were underpowered with regard to overall survival endpoints. For these reasons a majority of operable curative intent patients with locally advanced ESCC in the world are more likely to be treated with nCRT followed by surgery (12–14).

Consequently, a large, pragmatic and direct intention-to-treat comparison between the two guideline-recommended treatment options for operable ESCC, nCRT with mandatory surgery vs dCRT with surgery only when needed to achieve local tumor control, is still lacking and strongly warranted. This trial, named NEoadjuvant chemoradiotherapy for Esophageal squamous cell carcinoma versus Definitive chemoradiotherapy with salvage Surgery as needed (NEEDS), could potentially change the current globally dominating practice from neoadjuvant nCRT with subsequent mandatory esophagectomy, to dCRT with subsequent surveillance and surgical resection performed only when needed.

## Methods

### Study Objectives

#### Primary and Main Secondary Objective

The primary objective of the NEEDS trial is to demonstrate that dCRT with salvage esophagectomy as needed is non-inferior to nCRT followed by surgery regarding overall survival in patients with operable, locally advanced esophageal squamous cell carcinoma. The main secondary objective is to show superiority of dCRT with surgery only when needed, concerning global HRQOL one year after randomization

#### Secondary Objectives

- To study prespecified HRQOL endpoints relevant to esophageal cancer and effects of treatment for this disease, repeatedly during treatment and survivorship.
- To determine event free survival, loco-regional and distal relapse rates and histological response after chemoradiotherapy in the surgical specimen in the control arm.
- To investigate the overall health economic impact of each intervention. A cost-effectiveness analysis (CEA) will be conducted for all relevant endpoints comparing both interventions. Furthermore, the results of the trial-based CEA (e.g. patient-level resource use) will be used to build a decision analytic model and made publicly available to allow health care system-specific analysis by policy makers (e.g. country-specific costs or quality of life weights).
- To investigate the impact of each intervention on nutritional status during follow-up.
- To investigate whether any of the endpoints are affected by the type of radiological follow-up, CT or PET-CT, in patients treated with dCRT with salvage esophagectomy as needed.
- To investigate if there are any gender differences in any of the endpoints.
- To exploratively analyze putative tissue and liquid biomarkers in response to the different treatment strategies and long-term benefit. We aim to identify subgroups of patients who need trimodality treatment and those who have a good long-term outcome without esophagectomy.

### Trial Design

**Figure 1:**
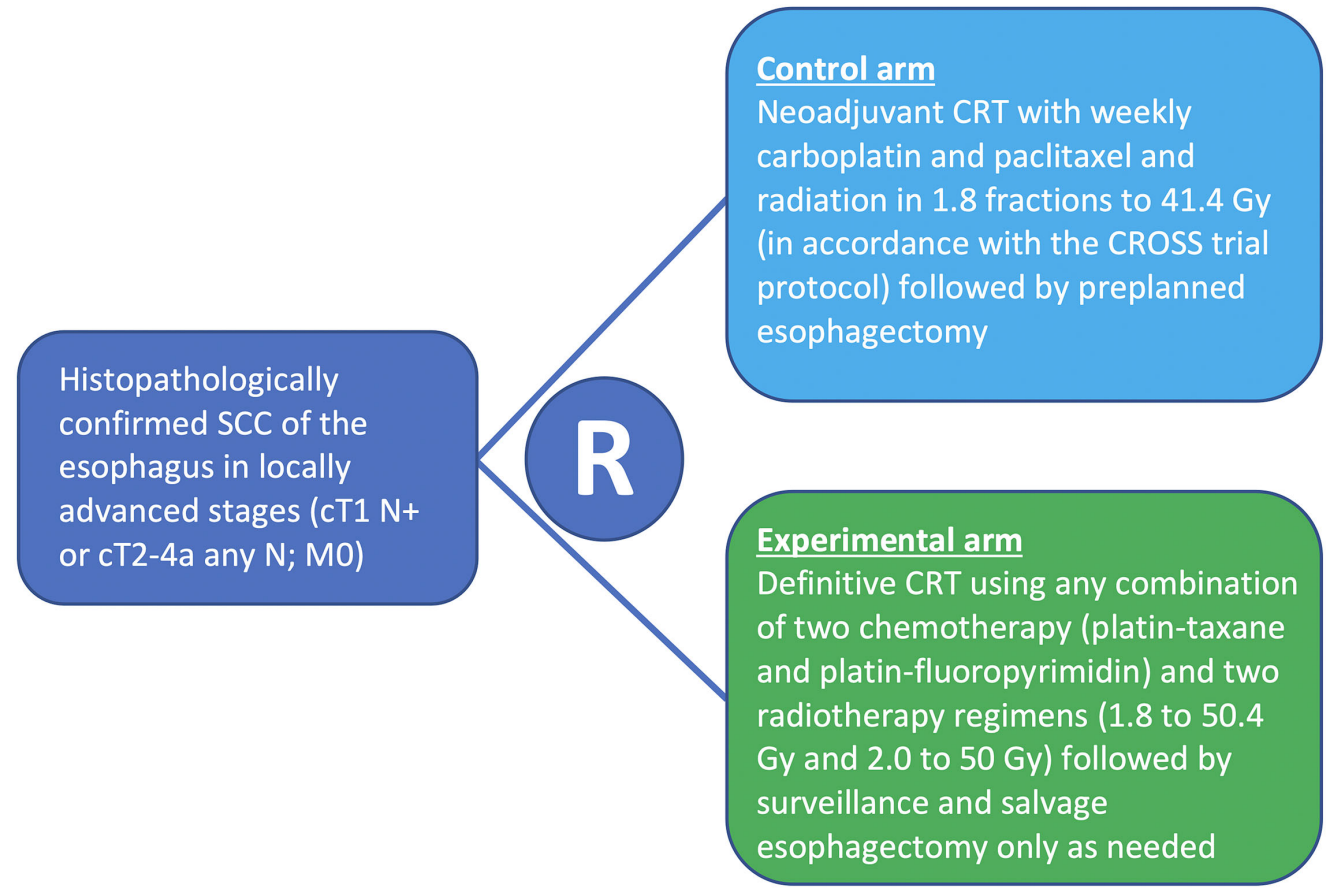
NEEDS randomization overview.

The NEEDS trial is a pragmatic, open-label, randomized controlled phase III multicenter trial. Randomization (1:1) of eligible patients is performed before any anti-tumor treatment is given (**Figure 1**).

### Study Setting

The NEEDS trial is an academic multicenter trial. To represent the global geographic incidence of ESCC the aim is to include patients from centers across most of the world´s continents, in order to maximize the generalizability of the results from the trial. Recruitment is so far planned in Sweden, Norway, Germany, Ireland, United Kingdom, France, Italy, Canada, India, China, Taiwan and Australia, and recruitment from several other countries are under discussion. The aim is to include around half of the patients in Asia and the other half in Western countries.

### Eligibility Criteria

#### Inclusion Criteria

- Histopathologically confirmed ESCC in locally advanced stages cT1 N+ or cT2-4a any N, M0, according to the current (8th) version of the AJCC TNM classification.
- Technically resectable disease according to the local multidisciplinary team conference (MDT)/tumor board.
- Age ≥ 18 years and ≤ 80 years.
- ECOG performance status 0-1.
  - Adequate organ function (assessed within 14 days prior to randomization):
  - White blood cell count (WBC) > 2 × 109/L.
  - Absolute neutrophil count (ANC) > 1.5 × 109/L.
  - Platelets ≥ 100 × 109/L.
  - Hemoglobin ≥ 9 g/dL.
  - Estimated glomerular filtration rate (eGFR) according to MDRD should be ≥ 60 ml/min.
  - Total bilirubin within normal limits (if the patient has documented Gilbert’s disease ≤ 1.5 * ULN or direct bilirubin ≤ ULN).
  - Aspartate transaminase (AST) and alanine transaminase (ALT) ≤ 1.5ULN.
- Women of childbearing potential (WOCBP) must have a negative serum or urine pregnancy test (minimum sensitivity 25 IU/L or equivalent units of HCG) within 24 hours prior to randomization. Furthermore WOCBP should undergo monthly pregnancy testing during the study treatment and for a period of up to six months after termination of the study treatment. Home pregnancy tests are acceptable.
- Patients of childbearing/reproductive potential should use highly effective method of birth control measures during the study treatment period and for at least *six* months after the last study treatment. A highly effective method of birth control is defined as those which result in low failure rate (i.e. less than 1% per year) when used consistently and correctly and include combined hormonal contraception associated with inhibition of ovulation (oral, intravaginal, transdermal), progestogen-only hormonal contraception associated with inhibition of ovulation (oral, injectable, implantable), intrauterine device, intrauterine hormone-releasing system, bilateral tubal occlusion, vasectomized partner, and sexual abstinence.
- Female subjects who are breast feeding should discontinue nursing prior to the first dose of study treatment and until *six* months after the last study treatment.
- Male study participants should not father children during the study treatment and for at least six months after the last dose and should use effective contraceptive measures throughout this time period. Effective contraceptive measures include condoms, vasectomy, and sexual abstinence. Male study participants who wish to be fathers in the future should be offered counselling on sperm storage before starting any study treatment.
- Absence of any psychological, familial, sociological or geographical condition potentially hampering compliance with the study protocol and follow-up schedule; those conditions should be discussed with the patient before registration in the trial.
- Before patient registration/randomization, written informed consent must be given according to ICH/GCP/GDPR and national/local regulations.

#### Exclusion Criteria

- M1 according to the current (8th) version of the AJCC TNM classification.
- cT4b according to the current (8th) version of he AJCC TNM classification.
- Primary tumor not resectable without laryngectomy.
- Impaired renal, hepatic, cardiac, pulmonary or endocrine status that compromises the eligibility of the patient for multimodality treatment with chemoradiotherapy followed by esophagectomy.
- Subjects not considered likely to tolerate multimodality treatment with chemoradiotherapy followed by esophagectomy.
- Subjects with previous malignancies are excluded unless a complete remission or complete resection was achieved at least 5 years prior to study entry. Adequately treated cervix uteri carcinoma in situ, and localized non-melanoma skin cancer are not exclusion criteria, regardless of time of diagnosis.
- Prior or concomitant treatment with radiotherapy or chemoradiotherapy with potential overlap of radiotherapy fields.
- Known uncontrollable hypersensitivity to the components of the chemotherapeutic agents used in the trial regimens.
- Inability to fully understand and digest information for study patients or to comply with study instructions due to language difficulty or cognitive failure such as dementia or severe psychiatric disorder.

### Interventions

#### Control Intervention (Arm A)

The control intervention is concomitant nCRT followed by esophagectomy in accordance with the intervention arm in the CROSS trial (4). Radiotherapy is administered 1.8 Gy × 23 (total dose: 41.4 Gy) as detailed below. Chemotherapy, using a platinum-taxane regimen, is administered starting on the first day of radiotherapy: carboplatin area under the curve (AUC) 2mg/ml/min + paclitaxel 50mg/m^2^ weekly × 5 (day 1, 8, 15, 22, 29).

Esophagectomy should be performed within 8 weeks of termination of chemoradiotherapy, unless the medical condition of the patient does not permit surgery within this timeframe.

##### Surveillance in Arm A

During the first two years, clinical status, symptoms and routine blood laboratory analyses (see below) will be followed up starting four to six weeks after surgery, then three months after surgery and then every third month up to two years after surgery. During years three to five after surgery, clinical status, symptoms and routine blood laboratory analyses (see below) will be followed every six months. Clinical follow-up visits can optionally be performed by telephone or using a video conference system. In addition, planned computerized tomography (CT) of neck, chest and abdomen will be performed at 9, 18, 36 and 60 months after surgery. The performance of PET-CT instead of CT is optional. Additional investigations should be performed on clinical suspicion of recurrence at any time. In the event of recurrence being diagnosed follow-up is terminated and the patient managed in accordance with local institutional treatment policy.

#### Experimental Intervention (Arm B)

The experimental intervention is concomitant dCRT, followed by surveillance, and esophagectomy only in case of residual or recurrent locoregional cancer. Radiotherapy can be administered using two alternative schemes, either 1.8 Gy × 28 (total dose: 50.4 Gy) or 2.0 Gy × 25 fractions (total dose: 50 Gy) as detailed below. Chemotherapy can be administered using any of the following regimens:

1. Platinum-Taxane Regimen (4)
  - Carboplatin AUC 2 mg/ml/min + paclitaxel 50mg/m^2^ on day 1 weekly during the full course of radiotherapy (5 or 6 weeks, depending on the radiotherapy regimen used).
2. Platinum-Fluoropyrimidine Regimens (5, 17)
  - Cisplatin 75mg/m^2^ on the first day of weeks 1 and 5 + 5-fluorouracil 1000 mg/m^2^/day by continuous infusion on the first four days of weeks 1 and 5.
  - FOLFOX: Oxaliplatin 85 mg/m^2^, calcium folinate 200 mg/m^2^ and 5-fluorouracil 400 mg/m^2^ on the first days of weeks 1, 3 and 5 + 5-fluorouracil 800 mg/m^2^/day by continuous infusion on the first two days of weeks 1, 3 and 5.

There is no induction chemotherapy or adjuvant chemotherapy.

##### Surveillance in Arm B

Clinical status, symptoms and routine blood laboratory analyses (see below) will be assessed 4 weeks after completed dCRT, then every 3 months up to two years after completed dCRT. From two years and up to five years after dCRT, clinical status, symptoms and routine blood laboratory analyses (see below) will be followed up every six months. Clinical follow-up visits can optionally be performed by telephone or using a video conference system. In addition a CT of the neck, chest and abdomen will be performed at 4 weeks after completed dCRT and at 3, 6, 9, 12, 15, 18, 21, 24, 30, 36, 42, 48, 54 and 60 months after terminated dCRT. The performance of PET-CT instead of CT is optional.

In addition, upper endoscopy with multiple bite-on-bite biopsies from the original tumor area and from suspected residual or recurrent tumor will be performed at 3, 6, 9, 12, 15, 18, 21, 24, 30, 36, 42, 48, 54 and 60 months after terminated dCRT. Endoscopic ultrasonography (EUS) with fine needle aspiration (FNA) of suspected malignant lymph nodes is optional. Additional investigations should be performed on clinical suspicion of residual or recurrent disease at any time. For example clinical suspicion of residual disease is warranted in patients with persistent severe dysphagia 6 weeks after termination of dCRT. In the event of distant metastatic recurrence radiological and endoscopic follow-up is terminated and the patient managed in accordance with local institutional treatment policy.

##### Salvage Esophagectomy

Salvage esophagectomy is indicated in patients allocated to the experimental intervention (arm B), if there is either histopathological evidence of residual or recurrent cancer, or radiological signs of disease progression from one post dCRT surveillance CT or PET-CT to a subsequent one (even without histopathological proof). The verdict of disease progression should be made at the local multidisciplinary team conference of the study center in question. Another prerequisite for salvage esophagectomy is that the patient should be considered physiologically fit for surgery. The decision of whether the patient is fit for surgery is left at the discretion of the responsible surgeon, following the regular procedure of operability assessment at each center based on conventional clinical assessment with optional support of any function tests considered useful.

### Radiotherapy and Dose Planning

#### Fractionation Schedule

The radiotherapy in the trial also follows a pragmatic approach. The radiotherapy in Arm A will be given in 23 fractions of 1.8 Gy, 5 fractions a week (once daily), to a total dose of 41.4 Gy. The overall treatment time should not exceed 39 days (five and a half weeks). The radiotherapy in Arm B will follow either of two schedules: 28 fractions of 1.8 Gy, 5 fractions a week (once daily), to a total dose of 50.4 Gy, with overall treatment time not exceeding 46 days or 25 fractions of 2 Gy, 5 fractions a week (once daily), to a total dose of 50 Gy, with treatment time not exceeding 42 days (six weeks).

#### Treatment Preparation

For treatment preparation the patients should undergo CT simulation in treatment position with a slice thickness of maximum 3 mm. A 4D-CT can be used according to local routines. The use of intravenous contrast is recommended. The gastric filling should be standardized at simulation and at each treatment fraction according to local routines. This can be accomplished for example by two hours fasting prior to simulation and treatment. Positioning of the patient will be according to local routines and immobilization devices should be in accordance with the departmental policy.

### Definition of target volumes

The principles of target delineation will be as follows: The gross tumor volume of the primary tumor (GTVT) and involved nodes (GTVN) should be delineated on a planning CT, taking all clinical, endoscopic, and radiological information (CT or PET-CT) into account. GTVT should include the entire circumference of the esophagus at the level of the tumor. The clinical target volume of the primary tumor (CTVT) includes a 3 cm margin in the cranio-caudal direction and 1 cm radial margin from GTVT with corrections for natural anatomic boundaries (such as heart, lungs, skeletal structures, kidneys, and liver) and oriented along the esophageal mucosa (not a simple geometric expansion). For tumors located in the gastro-esophageal junction a 2-cm distal margin of clinically uninvolved gastric mucosa is sufficient. The clinical target volume of involved nodes (CTVN) includes the GTVN plus a 0.5 cm margin in all directions, with corrections for natural anatomic boundaries. These two volumes should be joined to make the CTV (total), patchwork radiotherapy should be avoided, and different target volumes joined *via* the most probable lymphatic drainage. In case a 4D-CT or other robust motion management techniques are not available, the margins for motion management can be estimated to be 10 mm in the cranial and caudal direction and 3 mm in the radial directions and these should be included in the CTV (total), although the vertebral bodies should be entirely excluded. For proximal tumors, the cranial level of the CTV should not extend above the cricoid cartilage. The planning target volume (PTV) is applied according to local routines, commonly 5-10 mm. Organs at risk considered and delineated are the heart, the lungs, the spinal cord, and the kidneys.

In addition to these volumes patients in the experimental arm B will also be treated with elective lymph node irradiation (18). Elective lymph node irradiation will include the following regions for the respective tumor sites:

#### Proximal Tumors and Tumors Located in the Middle of Esophagus Mainly Above the Carina

Supraclavicular lymph nodes, analogous to level 4 in head and neck cancers (19) (**Supplementary Figure S1**). Levels 2-4 according to IASLC staging atlas (20) (**Supplementary Figure S2**) at the same levels as CTVT and CTVN: Paratracheal, pretracheal, mediastinal (anterior mediastinal, retrotracheal, posterior mediastinal and tracheobronchial), and para-esophageal lymph nodes.

#### Tumors Located in the Middle of Esophagus Mainly Below the Carina

Mediastinal, paratracheal, pretracheal and para-esophageal lymph nodes at the same levels as CTVT and CTVN, as well as paraaortic, paracardial, common hepatic, hepatogastric ligament, and celiac lymph nodes.

#### Distal Tumors

Paraaortic, paracardial, common hepatic, hepatogastric ligament, celiac, and para-esophageal lymph nodes (**Supplementary Figure S1**) at the same levels as CTVT and CTVN.

To encompass the para-esophageal lymph nodes, a radial margin of 1 cm from the outer esophageal wall is recommended.

### Treatment Delivery

Radiotherapy will be delivered using 3D conformal radiotherapy (3DCRT), intensity modulated radiotherapy (IMRT) or volumetric arc therapy (VMAT). Concerning the dose distribution, 98% of the PTV should receive at least 95% of the prescribed dose. However, if this objective is unachievable, 95% of the PTV should receive at least 95% of the prescribed dose. The maximum dose within the PTV should not exceed 107% of the prescribed dose.When planning the radiotherapy treatment, the dose constraints in **Table 1** should be adhered to. Treatment position verification should be done in accordance with individual departmental policy.

**Table 1:** Dose constraints.

Structure	Priority	Constraints	Description
**Spinal Cord**	1	D_0.1cc <_45 Gy	The dose given to 0.1 cm^3^ of the spinal cord should be less than 45 Gy. This constraint takes precedence over PTV coverage.
**Total kidneys**	2	V_18Gy <_30% D_mean_< 18 Gy	The volume receiving 18 Gy should be less than 30%. The mean dose should be less than 18 Gy. Both constraints take precedence over PTV coverage.
**Total lungs**	3	D_mean_< 20 Gy	The mean dose should be less than 20 Gy. This constraint takes precedence over PTV coverage.
	5	V_20Gy <_20%	The volume receiving 20 Gy should be less than 20%
**Heart**	4	V_30Gy <_30%	The volume receiving 30 Gy should be less than 30%.
		D_mean_< 30 Gy	The mean dose should be less than 30 Gy.

### Radiotherapy Modification for Treatment Delay

In case of missed treatment days, radiotherapy can be compensated at the discretion of the treating physician, though for patients randomized to Arm B (dCRT) the total radiation dose may be increased to a maximum EQD2 (equivalent total dose in 2Gy/fraction) of 55 Gy.

### Modifications for Toxicity

In the event of radiation induced grade 4 esophagitis, both chemotherapy and radiotherapy should be withheld until the esophagitis has recovered to grade 3. Alternatively, the treatment can be discontinued at the discretion of the treating physician. Dosing modifications for chemotherapy are provided in **Supplementary Table S1**.

### Radiotherapy Quality Assurance

The aim of the radiotherapy quality assurance (RT-QA) program is to ensure the consistency of radiotherapy treatment delivery across all participating centers as well as the verification of adherence to the protocol guidelines described above (21, 22). The RT-QA coordinating team is comprised of three medical physicists and three radiation oncologists based at the External Radiotherapy Department at the Karolinska University Hospital.

The RT-QA program is divided into two stages:

1. Pre-Study: All sites wishing to participate must submit a Facility Survey, perform a level 1 dosimetry audit, and complete two contouring benchmark exercises (one with a tumor located in the proximal and one in the distal esophagus) and two dose planning benchmark exercises with a proximal and a distal esophageal tumor respectively before patients can be included in the trial. These benchmark exercises must adhere to the radiotherapy guidelines described in the study protocol.
2. During study inclusion participating centers must send the radiotherapy data of each included patient to a specific online platform. The evaluation and monitoring procedure of the data is further divided into two levels. Level 1 consists of pre-treatment evaluation of contouring and dose planning of the first patient at each center. If major violations are identified in the contouring part, both re-delineation and re-planning will be requested. If violations are detected in the planning part, only re-planning will be requested. Once an acceptable quality level is achieved the centers progress to level 2. At this point, a retrospective evaluation of contouring and dose planning will be performed for one in every 5 patients, randomly selected, with feedback to centers regarding every evaluated patient.

In case of a loco-regional tumor recurrence (with or without simultaneous distant metastasis), the center is required to send the following DICOM data as soon as possible: diagnostic CT and, if available, the PET images, confirming the recurrence.

### Surgical Quality Assurance

All centers participating in this trial are high volume centers, with highly experienced surgeons. Prior to the trial, participating surgeons will be asked to provide their annual and life-time volume of esophagectomies performed for cancer. For each participating center information regarding 30-day and 90-day mortality over the preceding three years will be collected. Centers will before entering the trial be requested to complete a questionnaire, to describe their intended and most commonly used operative approach based on tumor location and staging that may be encountered within the trial. This will allow an assessment of expected degree of heterogeneity in surgical approach and expected lymphadenectomy within the trial. Standard quality outcome metrics including data on estimated blood loss and perioperative complications will be collected for every operation within the trial. Two still photographs of the lymphadenectomy fields upon completion of lymphadenectomy (abdomen and thorax) will be saved at each participating site for every operation. Specifically, the abdominal photograph should include the upper pancreatic border with the dissection of stations 7, 8a, 9 and 11p. The thoracic photo should include dissection of the subcarinal area. The surgery quality assurance committee will form consensus on grading lymphadenectomy from these photographs by collectively assessing the first three cases from ten centers. After this, a randomly selected 10% of the included patients (selection stratified by center and country) will be assessed by two of the members of the surgery quality assurance committee. The photos will be scored on a Likert scale with four levels (excellent, good, fair and poor).

### Outcomes

#### Primary and Main Secondary Endpoints

The primary endpoint of the NEEDS trial is overall survival (OS) with a minimum follow-up of two years. The main secondary endpoint is global HRQOL one year after randomization. The primary objective of treatment for localized esophageal cancer is cure and there are no known surrogate endpoints that can replace OS in a pragmatic trial aiming to establish best practice. However, the importance of patient reported HRQOL during survivorship qualifies HRQOL as a main secondary endpoint. The main secondary endpoint of HRQOL at one year after randomization will be the global HRQOL score in the EORTC QLQ-C30 questionnaire.

#### Secondary Endpoints

- HRQOL will be assessed at baseline, and thereafter 6, 12, 24, 36 and 60 months after randomization using the EORTC HRQOL Questionnaire QLQ-C30 version 3.0 (23),the EORTC HRQOL esophagogastric-specific questionnaire EORTC QLQ-OG25 (24) and the EQ5D questionnaire (25). Predefined endpoints will be global HRQOL, physical function, dyspnea, diarrhea, dysphagia and anxiety at all assessed time-points.
- Event free survival (EFS), defined as time to relapse, initiation of any anti-tumor therapy beyond the study treatments (salvage surgery is considered a study treatment in the dCRT arm), or death, whichever comes first.
- Loco-regional and distant relapse rates, including the relation of relapse location to the radiation field (in-field or out-field).
- Histopathological response according to Mandard (26), as well as other pathological data in operated patients, i.e. ypTNM including total and metastatic lymph node count, and tumor free resection margins, R0 (according to the Royal College of Pathologist’s definition of at least 1 mm without viable tumor cells) (27).
- Health economics will be assessed including patient-level medical resource use and societal costs due to sick-leave and other non-medical costs. Quality-adjusted life years (QALYs) will be calculated using EQ-5D, reported at baseline and 6, 12, 24, 36 and 60 months after randomization.
- Surgical complications according to the Esophagectomy Complications Consensus Group (ECCG) (28) and classified according to Clavien-Dindo (29) (Appendix 3)
- Treatment-related adverse events and toxicity coded by NCI CTCAE criteria version 5.0.
- Nutritional outcomes including weight change, dysphagia and appetite assessment.
- Gender stratified analyses of all endpoints.
- Exploratory analyses for putative tissue and liquid biomarkers for response to CRT and benefit from either of the two treatment strategies (optional per center).

### Follow-Up

**Figure 2:**
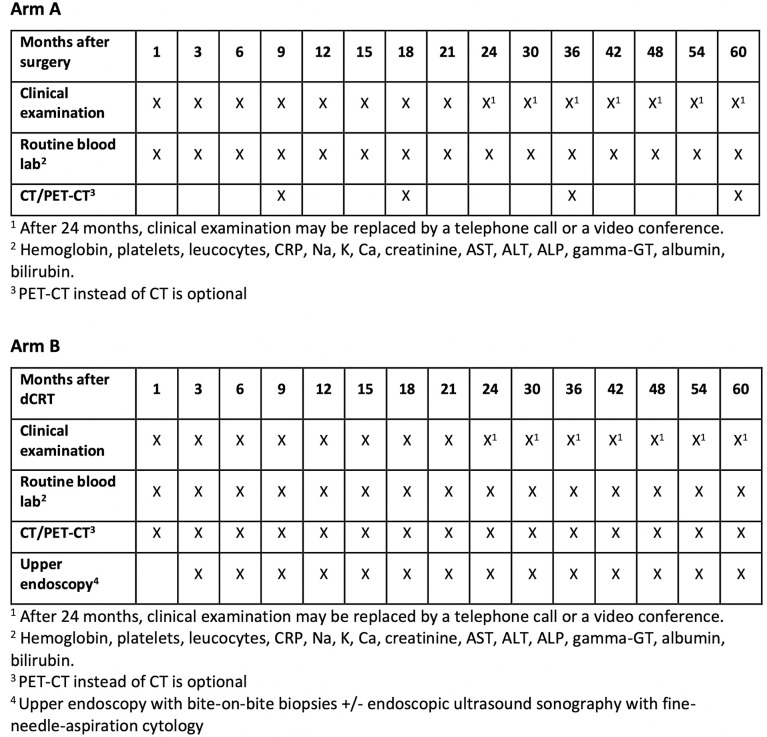
Overview of scheduled cancer recurrence surveillance by treatment allocation.

Included study subjects are followed up for five years. The follow-up and surveillance schemes for each study arm are described in detail above in the interventions section (**Figure 2**).

### Randomization and Stratification

Randomization is performed online from within an electronic randomization system once all eligibility criteria have been verified and no exclusion criteria have been fulfilled. Treatment allocation is open-label among the two arms in a 1:1 ratio. Randomization is performed using minimization schemes with stratification for gender (male or female), age (< 70 yrs or ≥70 yrs), tumor site (upper, middle or lower third of the esophagus), performance status (ECOG 0 or 1), clinical nodal status (positive or negative/unknown), clinical primary tumor status (T4a or any other T) and trial center. The electronic randomization module is accessed from all participating sites.

### Sample Size Calculation and Statistical Methods for Data Analysis

#### Sample Size Calculation

The study has a non-inferiority design to investigate whether dCRT followed by surveillance and salvage esophagectomy only as needed will not appreciably compromise mortality while potentially providing an improved HRQOL and reduced morbidity, compared to nCRT with planned esophagectomy. Based on data from the CROSS trial, the 5-year survival for patients receiving nCRT followed by surgery is 60% (6). For patients in the surveillance arm, a non-inferiority margin of 7.5% is considered to be clinically worthwhile as a trade-off for reduced surgical and post-surgical morbitity and improved HRQOL. The size of the non-inferiority margin was decided upon after discussions among professionals in the international esophageal cancer community, and representatives of the Swedish patient organization PALEMA. From clinical considerations, the assumption that surveillance would increase overall survival is remote and therefore a one-sided test for non-inferiority is assumed.

Assuming a 5-year accrual and 2-year follow-up duration, a sample size of 1200 patients will have 80% power with one-sided 95% confidence to declare non-inferiority with a margin of 7.5% (i.e. from 60% to 52.5% in the surveillance group) based on a 1:1 randomization. Based on these assumptions, the expected number of events is 462, corresponding to a hazard ratio of 1.26. The analysis will be according to the intention-to-treat (ITT) principle with no imputation for missing values.

Mean differences in HRQOL of 10 points or more will be considered clinically relevant.

### Statistical Methods

The primary endpoint analysis of OS will be performed on the ITT population using differences in the 5-year survival proportion between treatments estimated by the method of Kaplan-Meier. Further analyses will include proportional hazards regression modelling accounting for stratification factors providing estimates of relative differences between treatment groups. Results will be presented as a figure including point estimates of the treatment effect, lower and upper 90% confidence limits and the non-inferiority margin (HR). The trigger for analysis will be based on the data maturity of the trial at 5-years. This is obtained as the ratio of the variance of the pooled (by treatment) Kaplan-Meier curve at 5-yrs to the variance of the 5-year Kaplan-Meier estimate had all patients been followed for 5-years (30). For the study to be analyzed this ratio would need to exceed 90% (ideally 95%).

The full analysis set (ITT population) comprises all randomized patients.

Additional proportional hazards regression models accounting including potential confounders (age, gender, tumor stage, primary tumor position (proximal/mid/distal), type of dCRT) will be performed.

For the secondary endpoint HRQOL mean differences between the treatment arms will be estimated using linear mixed regression models. Results from these models will be presented as mean differences together with 95% confidence intervals.

## Discussion

The NEEDS trial is the first adequately powered randomized trial to compare the two guideline recommended treatments options for ESCC (11); nCRT followed by planned esophagectomy vs. dCRT with surveillance and salvage esophagectomy only when needed for locoregional tumor control. Due to the lack of high-level evidence that dCRT and surgery only as needed is non-inferior to nCRT with surgery regarding survival, dCRT is in many countries most often used in frail patients who may not tolerate surgery (14, 31, 32). For this reason, there is an apparent need for a pragmatic, international multicenter trial which can generate results with a high degree of generalizability. The results will have the potential to change the current practice of operating most patients, to a new global practice allowing organ preservation for most patients, with non-inferior survival and superior quality of life.

The field of ESCC treatment is moving fast. It is expected that both nCRT followed by planned esophagectomy and also dCRT will be complemented by immunotherapies, at least in defined subsets of patients. In the context of the approval of immunotherapy by EMA following the Checkmate-577 study (33), the implementation of adjuvant immunotherapy with Nivolumab in resected patients with pathological residual disease in the resection specimen is in line with the FDA and EMA approvals. The Study Steering Committee had its second meeting 29 October 2021. Immunotherapy was discussed again and the decision was to pragmatically allow immunotherapy according to the approval status of the drug and clinical practice regulations in each country. As overall survival will definitely be affected, with or without imbalance between the study groups, a decision was taken to consider amending the primary endpoint from overall survival alone to two primary endpoints in the form of overall survival and HRQOL, either as a composite or separate endpoints. This will be finally discussed and decided at one of the next steering committee meetings. In the next years we will also see novel trial results from studies assessing the implementation of immunotherapy into dCRT of localized ESCC such as Skyscraper-07 (NCT04543617) and Keynote-975 (NCT04210115).

Clinical trials are usually described as explanatory or pragmatic. Explanatory trials measure efficacy, meaning the benefit of the treatment under ideal conditions. The aim then being to establish whether the treatment works, and this type of trial is usually undertaken during the initial development phase of an intervention (34). In explanatory trials participants are usually carefully selected to be homogeneous and treatments are very carefully standardized in an ideal experimental situation in order to establish proof of principle (34). Although explanatory trials are necessary to test whether new interventions work, they have limited generalizability to clinical practice, where patients are heterogeneous, and treatment usually is less standardized (34). Pragmatic trials, such as the NEEDS trial, measure effectiveness, meaning the benefit of treatment in clinical practice, and should therefore take place in a routine healthcare, or clinical practice setting (34).

For the same reason, pragmatic trials should normally use ITT, rather than per protocol (PP) analysis for primary outcomes. In a trial of interventions given over a period of time (for example nCRT/dCRT, surveillance and surgery) patients defined as having received an intervention per protocol, are usually a selection of the most successfully treated, potentially introducing bias into PP analyses. Consequently, in the NEEDS trial all analyses will be based on the ITT principle, although for the primary outcome - OS - an additional PP analysis will also be performed.

In conclusion the NEEDS trial is a randomized, pragmatic phase III trial comparing today’s preferred standard of care, nCRT followed by planned esophagectomy, with the alternative therapy dCRT with surveillance and salvage esophagectomy only when needed for local tumor control, with a non-inferiority hypothesis for OS in locally advanced, operable ESCC. The objective is, if the study objectives of non-inferior OS and superior HRQOL are met, to guide a global change of practice from mandatory surgery to surgery only when really needed. The trial started inclusion in November 2020 and plans to include 1200 patients in several countries in Asia, Europe, Australia and North America, over the next 4-5 years.

## Data Availability Statement

The raw data supporting the conclusions of this article will be made available by the authors, without undue reservation.

## Ethics Statement

The studies involving human participants were reviewed and approved by Etikprövningsmyndigheten. The patients/participants provided their written informed consent to participate in this study.

## Author Contributions

MN, RW and FL developed the concept. All authors developed the various aspects of the protocol. MN, HO, GA and FL wrote the manuscript. All authors reviewed manuscript and approved the final version. All authors contributed to the article and approved the submitted version.

## Funding

The NEEDS trial is supported by a grant from the Swedish Research Council (grant number 2019-00423) aiming to cover central trial costs as well as overall trial costs in Sweden. Additional funding has been granted from the Swedish Cancer Society (grant number 200736) and from the Region of Stockholm (grant number 20200119). Local funding is being sought in all participating countries.

## Conflict of Interest

The authors declare that the research was conducted in the absence of any commercial or financial relationships that could be construed as a potential conflict of interest.

## Publisher’s Note

All claims expressed in this article are solely those of the authors and do not necessarily represent those of their affiliated organizations, or those of the publisher, the editors and the reviewers. Any product that may be evaluated in this article, or claim that may be made by its manufacturer, is not guaranteed or endorsed by the publisher.

## Supplementary Material

Supplementary Figure 1 Supraclavicular lymph nodes are analogous to level 4 in head and neck cancers. The cranial border is defined by the cricoid cartilage. The anterior borders correspond to the sternocleidomastoid muscles. Cranially the posterior border is the posterior edge of the sternocleidomastoid muscle. Caudally the posterior border is the anterior edge of the scalene muscles and the apex of the lungs. The medial limit is the medial edge of the common carotid artery. The lateral limit is defined by the lateral edge of the sternocleidomastoid muscle cranially and the lateral edge of the scalene muscles caudally. The inferior border extends into the thoracic inlet.

Supplementary Figure 2 Para-esophageal, paratracheal, pretracheal and mediastinal (anterior mediastinal, retrotracheal, posterior mediastinal and trachea-bronchial) lymph nodes. Above the carina, the CTV will encompass the entire trachea and extend radially to encompass the lower and upper paratracheal nodal stations which correspond to levels 2 and 4 in the IASLC staging atlas. Above the aortic arch the anterior border of the CTV is defined by the sternum and clavicular heads to encompass prevascular nodes (IASLC level 3). Above the level of the thoracic inlet, the trachea should be excluded from the CTV (unless the 1cm radial margin to the esophagus requires it). For distal tumors in which the CTV extends superiorly to the mediastinum only to respect the cranial margin to the primary tumor or to para-esophageal lymph node metastases, the superior nodal stations except for the para- esophageal lymph nodes need not to be included.

Supplementary Figure 3 Paraaortic, hepatogastric ligament, celiac lymph nodes. Below the diaphragm the CTV should be extended inferiorly to the level of the origin of the celiac axis. The CTV will be bounded in the lateral aspect by the vertebral body on the right (usually Th12) and 0.5-1 cm beyond the lateral aspect of the aorta on the left, the vertebral body posteriorly and the pancreatic body anteriorly. Between the level of the gastro- esophageal junction and the celiac lymph nodes the lesser curvature nodes will be included. In this region the liver will define the right border and the stomach will define the left border. Anteriorly the CTV includes the fatty space between the lesser curvature and the liver.

Supplementary Table 1 Guidelines for management of toxicity from chemotherapy graded according to CTCAE. *ULN, upper limit of normal.
