# Supplementary figures and images for: Neoadjuvant Chemoradiotherapy and Surgery for Esophageal Squamous Cell Carcinoma Versus Definitive Chemoradiotherapy With Salvage Surgery as Needed: The Study Protocol for the Randomized Controlled NEEDS Trial
Source: Front Oncol. 2022 Jul 13;12:917961. doi: 10.3389/fonc.2022.917961 (PMC9326032; doi:10.3389/fonc.2022.917961)

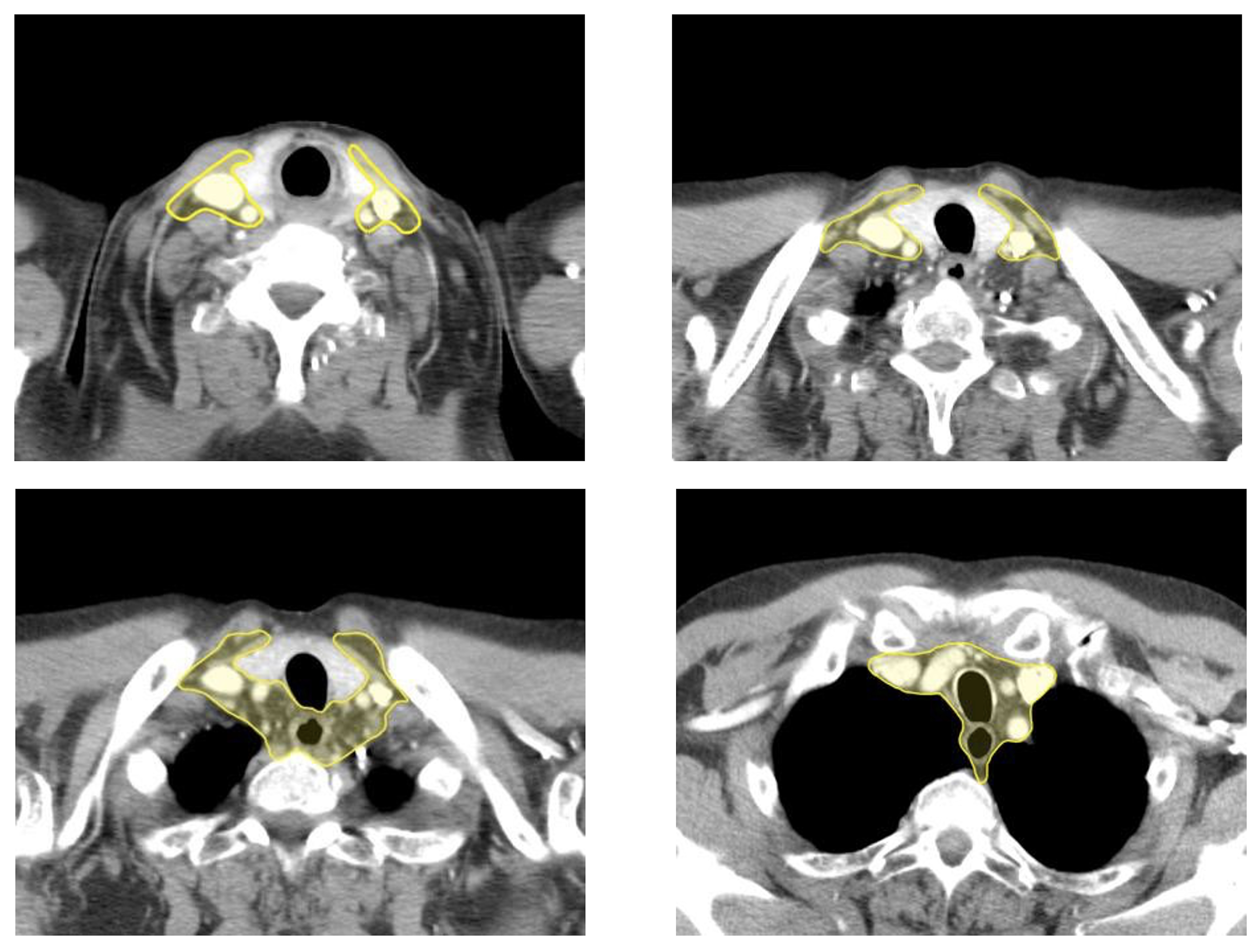

Supplement: Supplementary Figure 1 — Supraclavicular lymph nodes are analogous to level 4 in head and neck cancers. The cranial border is defined by the cricoid cartilage. The anterior borders correspond to the sternocleidomastoid muscles. Cranially the posterior border is the posterior edge of the sternocleidomastoid muscle. Caudally the posterior border is the anterior edge of the scalene muscles and the apex of the lungs. The medial limit is the medial edge of the common carotid artery. The lateral limit is defined by the lateral edge of the sternocleidomastoid muscle cranially and the lateral edge of the scalene muscles caudally. The inferior border extends into the thoracic inlet. [file Image_1.jpeg]

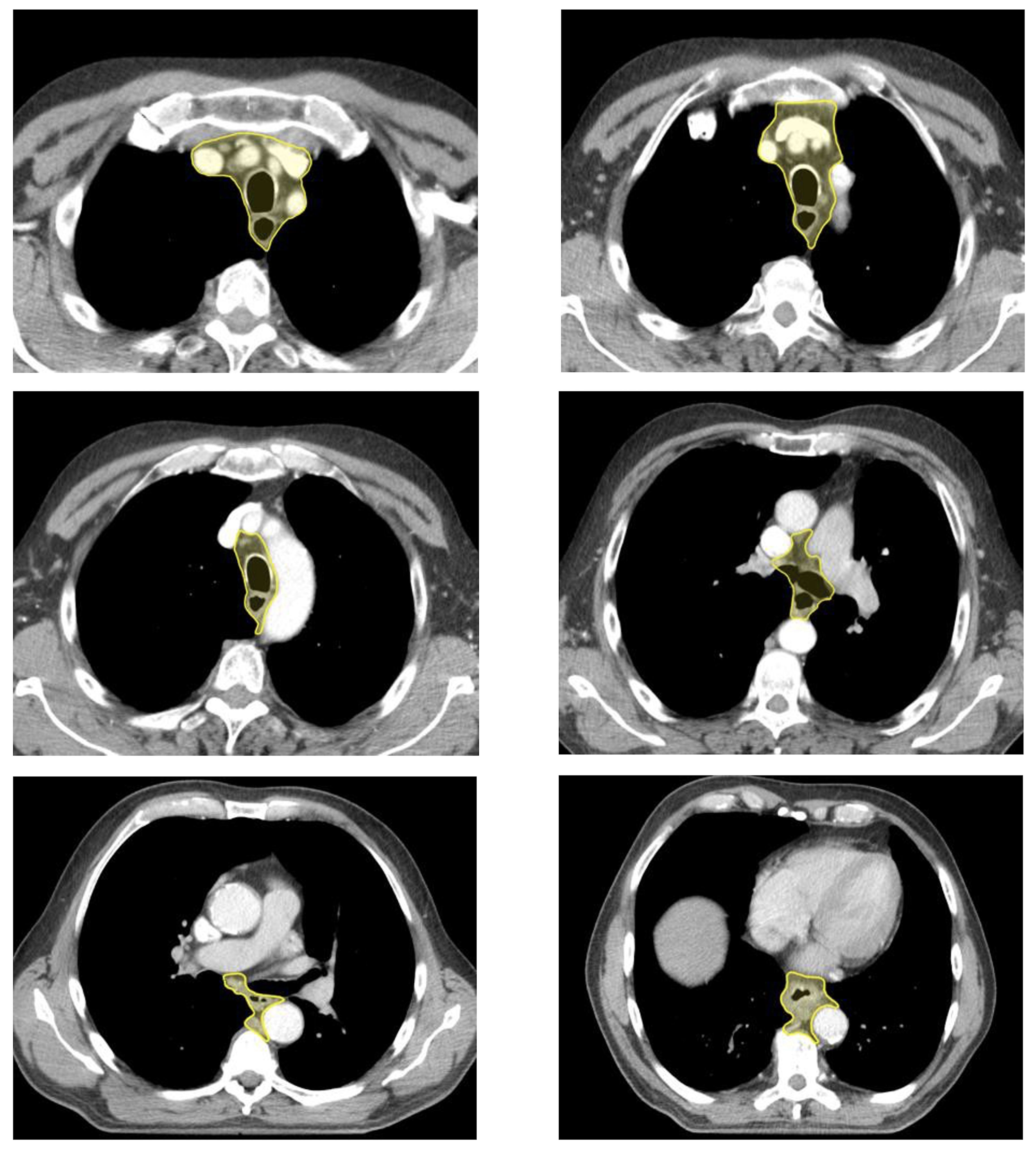

Supplement: Supplementary Figure 2 — Para-esophageal, paratracheal, pretracheal and mediastinal (anterior mediastinal, retrotracheal, posterior mediastinal and trachea-bronchial) lymph nodes. Above the carina, the CTV will encompass the entire trachea and extend radially to encompass the lower and upper paratracheal nodal stations which correspond to levels 2 and 4 in the IASLC staging atlas. Above the aortic arch the anterior border of the CTV is defined by the sternum and clavicular heads to encompass prevascular nodes (IASLC level 3). Above the level of the thoracic inlet, the trachea should be excluded from the CTV (unless the 1cm radial margin to the esophagus requires it). For distal tumors in which the CTV extends superiorly to the mediastinum only to respect the cranial margin to the primary tumor or to para-esophageal lymph node metastases, the superior nodal stations except for the para- esophageal lymph nodes need not to be included. [file Image_2.jpeg]

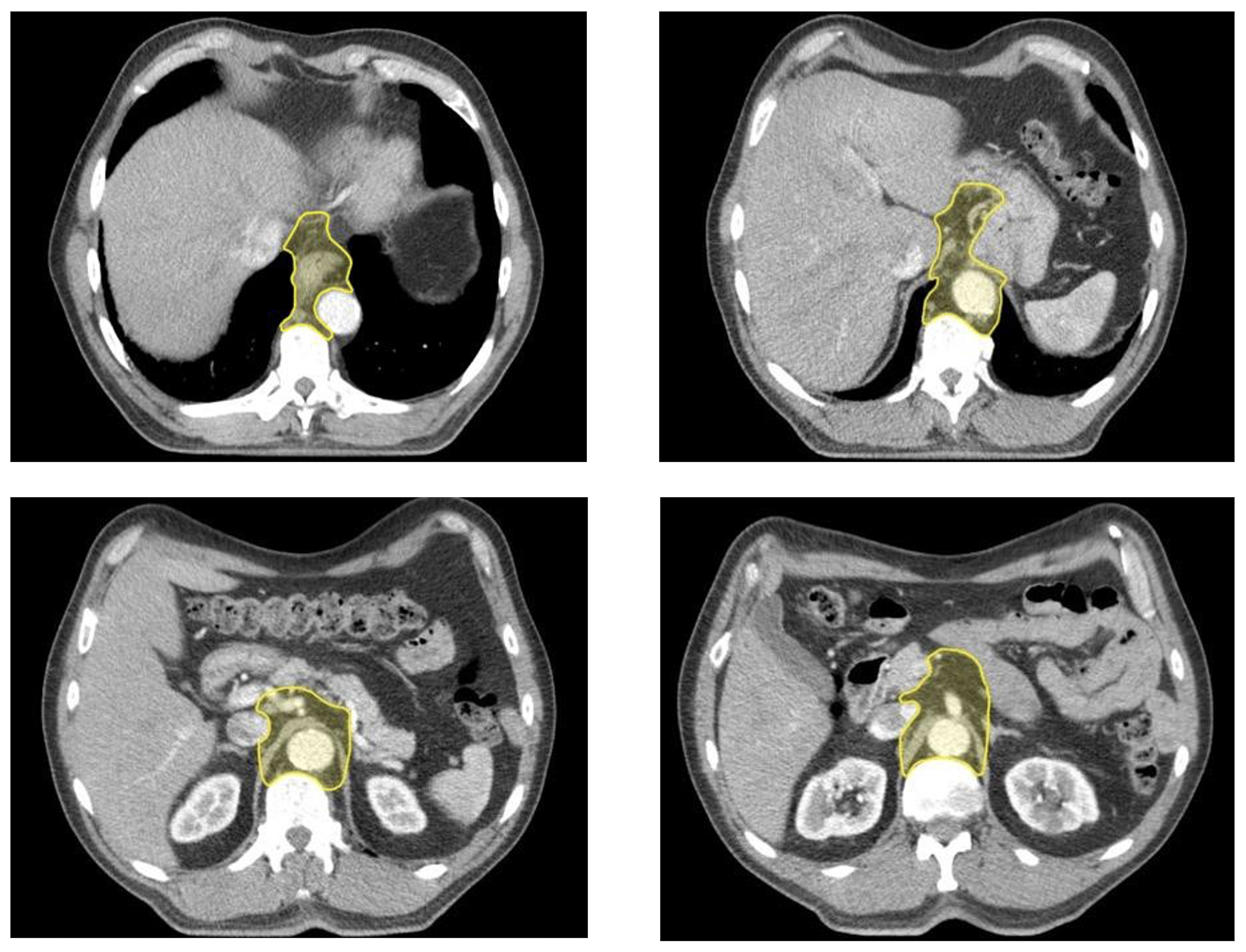

Supplement: Supplementary Figure 3 — Paraaortic, hepatogastric ligament, celiac lymph nodes. Below the diaphragm the CTV should be extended inferiorly to the level of the origin of the celiac axis. The CTV will be bounded in the lateral aspect by the vertebral body on the right (usually Th12) and 0.5-1 cm beyond the lateral aspect of the aorta on the left, the vertebral body posteriorly and the pancreatic body anteriorly. Between the level of the gastro- esophageal junction and the celiac lymph nodes the lesser curvature nodes will be included. In this region the liver will define the right border and the stomach will define the left border. Anteriorly the CTV includes the fatty space between the lesser curvature and the liver. [file Image_3.jpeg]
