# Supplementary material for: Neoadjuvant Chemoradiotherapy and Surgery for Esophageal Squamous Cell Carcinoma Versus Definitive Chemoradiotherapy With Salvage Surgery as Needed: The Study Protocol for the Randomized Controlled NEEDS Trial
Source: Front Oncol. 2022 Jul 13;12:917961. doi: 10.3389/fonc.2022.917961 (PMC9326032; doi:10.3389/fonc.2022.917961)
Supplement: Supplementary Table 1 — Guidelines for management of toxicity from chemotherapy graded according to CTCAE. *ULN, upper limit of normal. [file Table_1.docx]

**Supplementary Table 1: Guidelines for management of toxicity from chemotherapy graded according to CTCAE.**

| **Hematologic toxicity** | **Management** |
| --- | --- |
| **Weekly carboplatin/paclitaxel** |  |
| Neutropenia < 1.0x10^9^/l and/or  Thrombocytopenia < 75 x10^9^/l | Chemotherapy delayed until neutrophils are ≥ 1.0 x10^9^/l and platelets are ≥ 75 x10^9^/l.  Chemotherapy should be reduced by 75% from original dose for all subsequent cycles. |
| **FOLFOX regimen** |  |
| Grade 3 or 4 neutropenia and/or  Grade 3 or 4 thrombocytopenia | Chemotherapy delayed until neutrophils are ≥ 1.0 x10^9^/l and platelets are ≥ 75 x10^9^/l.  No bolus 5-fluorouracil is to be given and oxaliplatin is reduced to 65 mg/m^2^ for subsequent cycles. |
| **Cisplatin/5-fluorouracil regimen** |  |
| Grade 2 neutropenia and/or  Thrombocytopenia < 100 x10^9^/l | Cisplatin and 5-fluorouracil are to be reduced to 50%. |
| Grade 3 or 4 neutropenia | Chemotherapy is postponed for a week and G-CSF is to be given according to local routines after the next cycle. |
| Thrombocytopenia < 75 x10^9^/l | Chemotherapy is postponed for a week |
| **Non-hematologic toxicity** | **Management** |
| **Renal** |  |
| Creatinine ≤ 1.5 x ULN at the treatment day | Continue therapy. |
| Creatinine > 1.5 x ULN | Establish intravenous infusion the evening preceding treatment at a rate to correct any volume deficits and produce a urine flow ≥ 50 ml/h.  Repeat serum creatinine value in the morning:  ≤ 1.5 x the upper limit of normal → Proceed with treatment.  > 1.5 x the upper normal limit → Stop chemotherapy.  If cisplatin is the suspected cause of renal dysfunction, it can be replaced by oxaliplatin at the discretion of the treating physician |
| **Gastrointestinal** |  |
| Mucositis with oral ulcers or protracted vomiting despite antiemetic premedication | Delay chemotherapy one week |
| **Neurologic** |  |
| ≤ CTCAE grade 2 | Continue therapy |
| > CTCAE grade 2 | Stop chemotherapy |
| **Cardiac** |  |
| Asymptomatic bradycardia or isolated and asymptomatic ventricular extrasystoles | Continue therapy under continuous cardiac monitoring. |
| 1^st^ degree AV block | Continue therapy under continuous cardiac monitoring. |
| Symptomatic arrhythmia or AV block (except 1^st^ degree) or other heart blocks. | Stop paclitaxel, manage arrhythmia according to standard practice, patient goes off protocol. |
| **Other major organ toxicity** |  |
| CTCAE grade > 2 (except for esophagitis) | Stop therapy, patient goes off protocol treatment. |

*ULN = upper limit of normal
